# The impact of workplace violence on job satisfaction, job burnout, and turnover intention: the mediating role of social support

**DOI:** 10.1186/s12955-019-1164-3

**Published:** 2019-05-30

**Authors:** Xiaojian Duan, Xin Ni, Lei Shi, Leijing Zhang, Yuan Ye, Huitong Mu, Zhe Li, Xin Liu, Lihua Fan, Yongchen Wang

**Affiliations:** 1 Department of Health Management, School of Public Health, Harbin Medical University, Harbin, 150081 China; 2 Medical Dispute Office, Beijing Children’s Hospital, Capital Medical University, National Center for Children’s Health, Beijing, 100045 China; 3 Department of Psychiatry, the Fourth Affiliated Hospital of Harbin Medical University, Harbin, 150001 China; 4 Department of Medical Records, Dalian Children’s Hospital, Dalian, 116012 China; 5 Administrative Office, Harbin Children’s Hospital, Harbin, 150010 China; 6 Department of General Practice, the Second Affiliated Hospital of Harbin Medical University, Harbin, 150001 China

**Keywords:** Workplace violence, Job satisfaction, Burnout, Turnover intention, Mediating role, Social support

## Abstract

**Background:**

Workplace violence (WPV) is a global public health problem and has caused a serious threat to the physical and mental health of healthcare workers. Moreover, WPV also has an adverse effect on the workplace behavior of healthcare workers. This study has three purposes: (1) to identify the prevalence of workplace violence against physicians; (2) to examine the association between exposure to WPV, job satisfaction, job burnout and turnover intention of Chinese physicians and (3) to verify the mediating role of social support.

**Methods:**

A cross-sectional study adopted a purposive sampling method to collect data from March 2017 through May 2017. A total of nine tertiary hospitals in four provinces, which provide healthcare from specialists in a large hospital after referral from primary and secondary care, were selected as research sites based on their geographical locations in the eastern, central and western regions of China. Descriptive analyses, a univariate analysis, a Pearson correlation, and a mediation regression analysis were used to estimate the prevalence of WPV and impact of WPV on job satisfaction, job burnout, and turnover intention.

**Results:**

WPV was positively correlated with turnover intention (r = 0.238, *P* < 0.01) and job burnout (r = 0.150, *P* < 0.01), and was negatively associated with job satisfaction (r = − 0.228, *P* < 0.01) and social support (r = − 0.077, *P* < 0.01). Social support was a partial mediator between WPV and job satisfaction, as well as burnout and turnover intention.

**Conclusions:**

The results show a high prevalence of workplace violence in Chinese tertiary hospitals, which should not be ignored. The effects of social support on workplace behaviors suggest that it has practical implications for interventions to promote the stability of physicians’ teams.

**Trial registration:**

(Project Identification Code: HMUIRB2014005), Registered March 1, 2014.

**Electronic supplementary material:**

The online version of this article (10.1186/s12955-019-1164-3) contains supplementary material, which is available to authorized users.

## Background

Workplace violence (WPV) is a serious global public health problem and has attracted public attention [1, 2]. At the same time, WPV is inevitable in health service departments, and there is a universal belief that it is increasing [3, 4]. Previous studies have shown that healthcare workers are more likely to become victims of violence or aggression than other workers [1, 2, 5]. The World Health Organization (WHO) divides WPV into two types of violent behaviors: (1) physical violence (e.g., beating, kicking, slapping, stabbing, shooting, pushing, biting, and pinching) and (2) psychological violence (including verbal abuse, threats, etc.) [6]. One survey showed that the prevalence of workplace violence for emergency physicians in Morocco was 70% [7]. The prevalence of WPV for medical staff ranges from 50 to 88% in different countries [8–10]. The prevalence of physical violence suffered by nurses ranged from 18.22% in Ethiopia to 56% in Jordan; verbal abuse ranged from 63.8% in Korea to 89.58% in Ethiopia; and sexual harassment ranged from 13.02% in Ethiopia to 19.7% in Korea [11–14]. In China, according to a report from the Chinese Hospital Association, the proportion of hospitals that experienced WPV increased from 90% in 2008 to 96% in 2012 [15]. One study stated clearly that many physicians have been attacked by patients or visitors, or have been seriously injured or even killed from 2003 to 2013 [16]. A big data study on Chinese workplace violence showed that 290 cases of violent injuries in hospitals were reported by the Chinese media from 2000 to 2015, and the incidents of violent injuries were on the rise, mostly concentrated in the area of high-quality medical resources [17]. Most studies have shown that the occupational safety of Chinese physicians has reached a very critical juncture [17–19].

Research showed that workplace violence has an impact on medical personnel, hospitals, and society [20, 21]. Following instances in which doctors suffered workplace violence, this caused other issues such as reduced job performance [22], decreased job satisfaction [23], and negative effects in their own physical and mental health [24], which may increase turnover intention and influence their quality of life [19]. A study of emergency physicians in Turkey found a significant correlation between emotional exhaustion and total violence (*P* = 0.012) and verbal violence (*P* = 0.016); depersonalization and total violence (*P* = 0.021) and verbal violence (*P* = 0.012) also have a significant correlation [25]. Physical violence and bullying were related to turnover intentions and lower job satisfaction [23]. A study showed that WPV has a significant effect on burnout and turnover intention [26]. WPV has severely interfered with the normal medical order in hospitals, which has caused many negative effects for medical services [27].

Previous studies have found that social support can alleviate the impact of violence on health and work-related outcomes (such as high anxiety, work stress, and dissatisfaction) [28, 29], thereby reducing the adverse consequences for medical staff who experience WPV. Although researchers have a variety of definitions for social support, the shared characteristic is the connection between the availability of external resources with social relationships. Social support can be divided into two categories. One type consists of objective, visible, or practical support, including direct material aid, social networks and the existence and participation of group relationships (such as family, spouses, friends and colleagues). The other type is subjective and experiential emotional support, which refers to the emotional experience and satisfaction of individuals who are respected, supported and understood in the society, and is closely related to the subjective feelings of the individual. Woodhead and other scholars have found that work resources (support from supervisors, friends, or family members, and the cultivation of opportunities) are associated with less emotional exhaustion and higher levels of personal achievement [30]. For employees with low levels of peer support, the negative relationship between unsafe working conditions and low work commitment was stronger [31]. Psychological violence at work was associated with low levels of workplace emotional commitment and high levels of turnover intention. Social support plays an intermediary role in the relationship between workplace violence and turnover intentions [32]. Social support plays a role in mitigating or mediating the impact of WPV on job burnout [33]. Brough’s findings indicated that managers’ support alleviated the psychological pressure of medical assistants who experienced verbal violence [34]. These previous empirical studies have demonstrated that social support plays a buffering or mediating role in the impact of WPV on work-related outcomes. Moreover, social support theory often focuses on vulnerable groups (such as battered women) [35]. Healthcare workers are considered vulnerable groups [36], therefore, we conducted research under the framework of social support. In summary, it is of great practical significance to study the roles of social support on the effects of WPV and workplace behaviors for the stability of hospital physicians. On the other hand, the development of this study is conducive to promoting the healthcare system in China to pay more attention to WPV and establish a unified WPV reporting system as soon as possible. The classical mediation test used the causal stepwise regression method [37], while the bootstrap test was used to re-examine the role of social support in workplace violence and workplace behavior [38]. Thus, to better understand the role of social support in workplace violence and physicians’ job satisfaction, job burnout, and turnover intention, we proposed the following hypotheses:

Hypothesis 1: WPV in hospitals is related to physicians’ job satisfaction, job burnout, and turnover intention; and.

Hypothesis 2: Social support plays a mediating role in WPV physicians’ job satisfaction, job burnout and turnover intention.

## Methods

### Study design and population

A cross-sectional survey was conducted based on the geographical location of the eastern (Beijing), middle (Heilongjiang, Anhui) and western (Shaanxi) regions in China. Nine tertiary public hospitals were selected as survey sites mainly with the assistance of China Hospital Association. The purposive sampling method was used to conduct a cross-sectional survey of physicians. These hospitals were similar in size, department setting and number of physicians. Therefore, the total number of physicians in nine hospitals is about 18,450; a total of 1486 samples were extracted, with these physicians accounting for 8.05% of the total. On average, 225 physicians from each hospital were extracted. All investigators conducted uniform training before starting investigations, and served in the role of investigator only after passing the assessment. Before the formal investigation, four hospitals in Harbin City were selected as our pre-surveyed sites, and 200 questionnaires were issued and collected. This part of the data was not included in the final analysis, because after processing the preliminary data, we further modified the questionnaire. Finally, we consulted health management experts, hospital administrators, clinicians, and other health experts (a total of six experts) on the questionnaire, and based on their feedback, we developed the final field survey questionnaire.

### Data collection

The survey was conducted from March 2017 through May 2017. Before the investigation, we communicated and coordinated with each research hospital and started the field investigation after obtaining consent. Members of the research group distributed questionnaires at the scene, and the respondents completed the anonymous questionnaires on the same day and returned them to the designated box. The hospital manager did not supervise the whole process. A total of 1486 questionnaires were issued to physicians and 1257 valid questionnaires were recovered (the effective response rate was 84.59%). The following conditions were selected as the inclusion criteria for this study: 1) possessing a professional doctors’ certificate; 2) having at least one year of clinical experience; 3) being engaged in clinical work during the investigation; and 4) voluntary participation without prejudice to the participants’ work.

### Final version of questionnaire

#### Demographic characteristics

The demographic information of physicians was collected, including gender, age group, marital status, educational background, professional title, employment form, department, years of experience and daily working hours.

#### WPV scale

In this study, a new measurement tool developed by Chen was used to evaluate the prevalence of WPV in the past year [39]. The WPV was divided into three dimensions (verbal violence, physical violence and sexual harassment) and nine items (which can be seen in the Additional file 1: Questionnaire). Verbal violence includes verbal attacks (insulting, degrading, or using other words that harm personal dignity, whether in face-to-face encounters or through phone calls, letters, networks, or leaflets, etc.), but no physical contact; threats (involving personal and property safety, as well as threatening complaints), but no physical contact. Physical violence includes physical contact or an attack with an object (including hitting, kicking, slapping, stabbing, pushing, biting, throwing objects, twisting arms, pulling hair, etc.), and sexual harassment/violence (sexual assault, rape or attempted rape). These behaviors were very common issues for Chinese physicians at work. Most importantly, these behaviors of mistreatment completely meet the standards for the definition and scope of WPV. The score for each item was four points, which reflected the frequency of respondents’ exposure to WPV (0 = zero times, 1 = 1 time, 2 = 2 or 3 times, and 3 = more than 3 times). The total possible score ranged from 0 to 27, and the higher the total score, the higher the frequency of violence exposure in the workplace. The scale had good reliability and validity, and has been widely used in China [2, 19, 40]. The Cronbach’s coefficient of this scale in this study was 0.871.

#### Social support rating scale

Social support was evaluated using the Chinese version of the Social Support Rating Scale (SSRS) [2, 41, 42], which is a brief measure of the respondents’ social support after experiencing WPV. This 10-item scale was divided into three dimensions: subjective support (three items), objective support (four items) and utilization of support (three items). Subjective support refers to an individual’s emotional experience of being respected, supported, and understood by their social group. Objective support refers to objective, visible, or actual support, including direct material support, support from social networks, and the existence and participation of group relationships, etc. The degree of utilization of social support refers to differences in the use of social support by individuals. The present study revealed that Cronbach’s α for SSRS was 0.875, and for the three subscales, it was 0.873 (subjective support), 0.886 (objective support) and 0.842 (utilization of support).

#### Minnesota job satisfaction short scale

The Minnesota Satisfaction Questionnaire Short Scale (MSQ-SS) was used to assess participants’ job satisfaction after experiencing WPV. [43, 44]. It includes two subscales (intrinsic satisfaction and external satisfaction), with a total of 20 items. The intrinsic satisfaction scale contains 12 items and the extrinsic satisfaction scale includes eight items. The general satisfacttion scale was formed by all items. These 20 items measured the respondent’s satisfaction with their competency development, sense of accomplishment, activities, promotions, empowerment, company policies and practices, compensation, co-workers, creative freedom, social services, social status, management–employee relations, management’s skills, diversification in work, and working conditions. Each item was divided into five levels, (1 = strongly unsatisfied, 2 = unsatisfied, 3 = uncertain, 4 = satisfied, and 5 = strongly satisfied). The higher the self-evaluation of the participants, the higher their satisfaction with the work. This study showed that the Cronbach’s coefficient for the MSQ-SS is 0.894, and for the two subscales, it is 0.902 (internal satisfaction) and 0.876 (external satisfaction).

#### Maslach burnout inventory—general survey

In this study, burnout was measured using the Maslach Burnout Inventory—General Survey (MBI-GS), and there were a total of 15 items [45, 46]. Each item of MBI-GS was rated based on the frequency of responder’s working experience, using 7 points of self-assessment, where 0 is never, and 6 is daily. MBI-GS is divided into three subscales to reflect job burnout, including emotional burnout, depersonalization, and reduced personal accomplishment. The first two subscales adopted the positive scoring, that is, the higher the score, the more serious the burnout. However, personal achievement was a reverse scoring system, that is, as the score becomes lower, the burnout is more serious. The scores of the three subscales are equal to the average of the sum of the items of each subscale. The total score was calculated from the scores of the three subscales, ranging from 0 to 18 points. The higher the score, the higher the burnout level. In this study, the Cronbach’s coefficient of MBI-GS was 0.872. The internal consistency coefficients were 0.834, 0.826, and 0.812, respectively, which were emotional exhaustion, depersonalization, and reduced personal accomplishment.

#### Turnover intention scale

This study used the turnover intention scale to measure nurse’ turnover intention after experiencing WPV. The scale was compiled by Michael and Spector [47] and revised by Lee G and Lee D [48], and included 6 items. The scale was divided into three dimensions: the possibility of employees resigning, the motivation of employees to find other jobs, and the possibility of employees obtaining external work. Each item reflected the number of times the participant had an intention to leave, and it was divided into four levels (1 = never, 2 = very few, 3 = occasionally, 4 = often). The total possible score was calculated by adding scores for all items, and it ranged from six to 24 points, with a higher score indicating a stronger intention to leave. A total average score ≤ 1 indicates that turnover intention is particularly low, low when it is from 1 to 2, higher when it is from 2 to 3, and exceptionally high when it is greater than 3. In this study, Cronbach’s α of turnover intention scale was 0.856.

### Data analysis

IBM SPSS V.19.0 was used for the data analysis in this study. The demographic characteristics of respondents were collected to report sample information. We used independent sample t-tests or one-way analysis of variance to compare group differences on the measurements of the continuous variables. The Pearson correlation was assessed for WPV, job satisfaction, burnout, and turnover intention. The regressions including mediations were calculated with the SPSS PROCESS macro by Preacher and Hayes [38]. The mediation analyses were based on model number 4 and bootstrapping (1000 bootstrap samples) using 95% confidence intervals. Statistically significant variables in univariate analysis are included as covariates: WPV as an independent variable (X); social support as an M variable; and job satisfaction, job burnout and turnover intention as dependent variables (Y). The macro allows calculating and testing the direct effect, the total effect, and the indirect effect. The effect is significant when the 95% CI does not include 0. Based on the bootstrap mediation effect test, there are two steps. First, we test whether a*b is significant. If a*b is significant, we need to test the positive and negative of a*b*c′. If a*b*c′ is positive, it is a complementary mediation. All study variables were tested for multicollinearity. A *P*–value < 0.05 was considered statistically significant.

## Results

### Demographics and characteristics of hospitals

Of the 1257 respondents who met our inclusion criteria, 53.6% were men, 56.6% received a postgraduate education, and 74.9% were married. The demographic characteristics of the participants are shown in Table 1. The common feature of these nine tertiary hospitals is that the number of beds is more than 500. Moreover, these hospitals are medical prevention technology centers with comprehensive medical, teaching, and scientific research capabilities.

**Table 1:** Demographic characteristics of the whole sample (*N* = 1257)

Variables	*n*	Percentage(%)
Gender		
Men	674	53.6
Women	583	46.4
Age group (years)		
≤ 30	345	27.4
31–50	813	64.7
≥ 51	99	7.9
Education level		
Junior college or below	44	3.5
College	502	39.9
Master or above	711	56.6
Marital status		
Married	942	74.9
Single/divorced/widowed	315	25.1
Professional title		
Primary	462	36.8
Intermediate	477	37.9
Senior	318	25.3
Department		
Internal medicine	444	35.3
Surgery	351	27.9
Obstetrics and Gynecology	59	4.7
Pediatrics	70	5.6
Department of stomatology	28	2.2
Otorhinolaryngology	71	5.6
Auxiliary examination	65	5.2
Other	169	13.5
Employment forms		
Formal staff	815	64.8
Appointment staff	442	35.2
Years of experience		
≤ 4	416	33.1
5–10	372	29.6
≥ 11	469	37.3
Daily working hours		
≤ 8	88	7.0
8–12	1032	82.1
≥ 12	137	10.9

### Prevalence of different styles of WPV against physicians

About 66.19% (832/1257) of participants reported having experienced WPV within the past 12 months. The 65.31% (821/1257) of physicians experienced verbal violence, which was the highest incident rate among all kinds of WPV in hospitals. During the previous 12 months, the prevalence of physical violence and sexual harassment toward physicians was 12.57% (158/1257) and 0.88% (11/1257), respectively. The respondents reported that the patients’ relatives were the main perpetrators (54.2%, 451/832), followed by the patients (26.4%, *n* = 220/832).

### Correlations between study variables

As shown in Table 2, all variables were significantly correlated with each other. The average frequency of WPV was 2.31 times in the last year. The average scores of job satisfaction, job burnout and turnover intention were 3.47, 6.30, and 14.09 points, respectively. WPV was positively correlated with turnover intention (r = 0.238, *P* < 0.01) and job burnout (r = 0.150, *P* < 0.01). WPV was negatively associated with job satisfaction (r = − 0.228, *P* < 0.01) and social support (r = − 0.077, *P* < 0.01).

**Table 2:** The Pearson correlation analysis among research variables

Variables	M ± SD	1	2	3	4
Workplace violence	2.31 ± 2.67	1			
Turnover intention	14.09 ± 3.76	0.238^**^	1		
Job satisfaction	3.47 ± 0.57	−0.228^**^	− 0.410^**^	1	
Job burnout	6.30 ± 3.01	0.150^**^	0.423^**^	−0.491^**^	1
Social support	41.03 ± 7.71	−0.077^**^	−0.199^**^	0.312^**^	−0.272^**^

^**^P < 0.01

### The difference between participants’ characteristics and scores of multiple variables

There was a significant difference in the scores on job satisfaction depending on the physicians’ demographics, including their age group, marital status, different professional titles, form of employment, department, years of experience, and daily working hours. The descriptive association between respondents’ characteristics and the burnout, workplace violence, social support, and turnover intention scores can be seen in Table 3.

**Table 3:** Univariate analysis and description of each scale

Characteristics	Workplace violence	Job satisfaction	Burnout	Social support	Turnover intention
	M ± SD	M ± SD	M ± SD	M ± SD	M ± SD
Gender					
Male	2.37 ± 2.63	3.48 ± 0.56	6.28 ± 2.96	41.15 ± 7.82	14.01 ± 3.79
Female	2.23 ± 2.75	3.45 ± 0.57	6.32 ± 3.07	40.90 ± 7.60	14.18 ± 3.73
F/t	0.905	1.097	−0.254	0.565	−0.972
Age group (years)					
≤ 30	1.83 ± 2.42	3.57 ± 0.60	6.52 ± 3.00	38.50 ± 7.06	13.79 ± 3.87
31–50	2.49 ± 2.74	3.43 ± 0.54	6.30 ± 2.98	41.89 ± 7.74	14.33 ± 3.65
≥ 51	2.42 ± 2.93	3.43 ± 0.61	5.51 ± 3.15	42.79 ± 7.74	13.18 ± 4.11
F/t	7.443^**^	8.419^**^	4.414^*^	27.289^**^	5.603^**^
Level of education					
Below undergraduate	2.34 ± 3.46	3.49 ± 0.70	5.78 ± 3.48	39.41 ± 10.57	13.82 ± 3.78
Undergraduate	2.11 ± 2.53	3.51 ± 0.53	6.02 ± 2.94	41.26 ± 7.68	13.76 ± 3.79
Master’s degree or above	2.44 ± 2.74	3.44 ± 0.58	6.53 ± 3.01	40.98 ± 7.53	14.34 ± 3.73
F/t	2.291	2.155	5.010^**^	1.203	3.643^*^
Marital status					
Married	2.42 ± 2.72	3.45 ± 0.56	6.18 ± 3.04	42.28 ± 7.48	14.18 ± 3.73
Single/ divorced/ widowed	1.87 ± 2.53	3.53 ± 0.57	6.68 ± 2.86	37.25 ± 7.24	13.90 ± 3.82
F/t	3.096^**^	−1.969^*^	−2.467^*^	10.159^**^	1.122
Professional title					
Primary title	2.50 ± 3.11	3.55 ± 0.56	6.36 ± 3.02	39.18 ± 7.49	13.84 ± 3.67
Intermediate title	2.43 ± 2.54	3.42 ± 0.55	6.34 ± 3.04	42.07 ± 7.61	14.49 ± 3.74
Senior title	2.04 ± 2.50	3.42 ± 0.59	6.16 ± 2.95	42.18 ± 7.69	13.86 ± 3.89
F/t	3.589^*^	7.177^**^	0.451	21.846^**^	4.351^*^
Employment form					
Long-term employee	2.43 ± 2.76	3.44 ± 0.57	6.36 ± 3.06	41.54 ± 8.00	14.16 ± 3.87
Temporary employee	2.09 ± 2.54	3.52 ± 0.56	6.19 ± 2.92	40.10 ± 7.07	13.96 ± 3.55
F/t	2.151^*^	−2.503^*^	0.986	3.170^**^	0.909
Department					
Internal medicine	1.96 ± 2.29	3.43 ± 0.59	6.57 ± 3.04	40.45 ± 7.48	14.20 ± 3.81
Surgery	2.62 ± 2.84	3.46 ± 0.57	6.63 ± 3.03	40.62 ± 8.21	14.32 ± 3.91
Obstetrics and Gynecology	3.25 ± 4.52	3.35 ± 0.59	6.64 ± 2.87	40.34 ± 7.96	14.19 ± 3.57
Pediatrics	2.56 ± 2.05	3.62 ± 0.51	5.54 ± 2.69	42.90 ± 7.61	14.69 ± 3.25
Department of stomatology	1.75 ± 2.14	3.55 ± 0.59	6.03 ± 3.10	43.89 ± 7.03	15.07 ± 4.91
Otorhinolaryngology	2.37 ± 2.52	3.40 ± 0.51	5.43 ± 2.91	40.97 ± 7.40	14.96 ± 3.15
Auxiliary examination	2.32 ± 2.66	3.65 ± 0.54	5.49 ± 2.85	43.23 ± 7.27	12.35 ± 3.91
Other	2.21 ± 2.74	3.51 ± 0.54	5.82 ± 2.96	41.59 ± 7.34	13.20 ± 3.23
F/t	3.316^**^	2.689^**^	4.113^**^	2.612^*^	4.732^**^
Years of experience					
≤ 4	1.83 ± 2.51	3.53 ± 0.56	6.57 ± 2.98	38.53 ± 7.52	13.73 ± 3.78
5–10	2.37 ± 2.57	3.44 ± 0.53	6.28 ± 3.00	41.07 ± 7.77	14.47 ± 3.64
≥ 11	2.68 ± 2.87	3.43 ± 0.59	6.08 ± 3.03	43.22 ± 7.17	14.11 ± 3.82
F/t	11.465^**^	3.747^*^	2.962	43.561^**^	3.824^*^
Daily working hours					
≤ 8	1.97 ± 3.30	3.61 ± 0.57	5.23 ± 2.66	41.49 ± 8.51	12.66 ± 3.18
8–12	2.20 ± 2.48	3.47 ± 0.56	6.25 ± 2.99	41.06 ± 7.53	14.10 ± 3.76
≥ 12	3.34 ± 3.48	3.35 ± 0.62	7.39 ± 3.09	40.55 ± 8.56	14.92 ± 3.89
F/t	11.761^**^	5.703^**^	15.043^**^	0.421	9.843^**^

^*^P < 0.05, ^**^ *P* < 0.01

### Mediation regression models of study variables

**Fig. 1:**
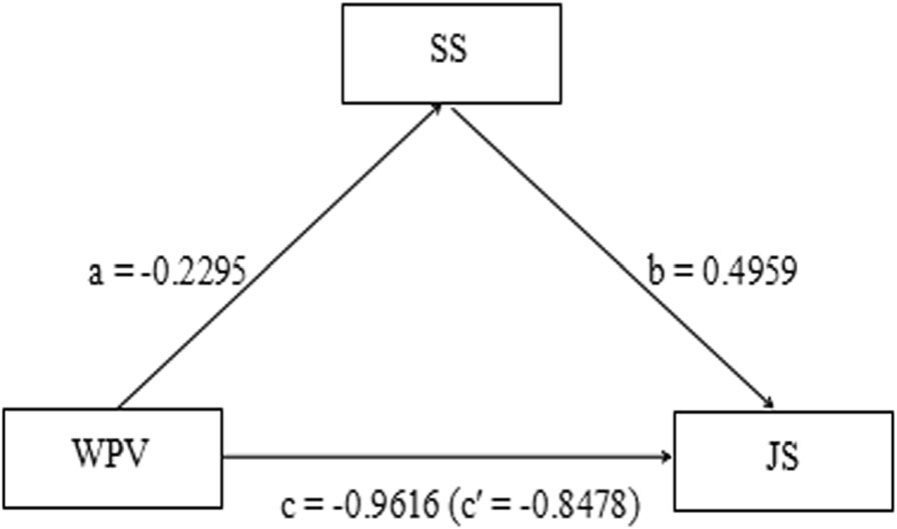
The mediating role of SS in explaining the relation between the WPV and JS of physicians (path 1 in Table 4). *N* = 1257; controlled for age, marital status, professional title, employment form, department, years of experience, and daily working hours; WPV, workplace violence; JS, job satisfaction; SS, social support; a = direct effect of WPV on mediator; b = direct effect of mediator on JS; c = total effect of WPV on JS; c′ = direct effect of WPV on JS

To give a brief overview, let us take Path 1 (seen in Table 4) as an example. The results of Table 4 and Fig. 1 can be summarized as follows: the direct effect of workplace violence on social support is − 0.2295, the direct effect of social support on job satisfaction is 0.4959, direct effect of WPV on job satisfaction is − 0.8478, and the total effect of WPV on job satisfaction is − 0.9616. Meanwhile, a*b*c′ is positive, showing the mediating role of social support in explaining the relation between the WPV and job satisfaction of physicians (Fig. 1). Similarly, Paths 2 and Path 3 indicate the mediating role of social support (Table 4).

**Table 4:** Results of mediation analyses

Paths	a	b	c’	a*b	95% CI of a*b	c	SE	R^2^
WPV→ SS → JS	− 0.2295^**^	0.4959^**^	− 0.8478^*^	− 0.1138^*^	(− 0.2079, − 0.0429)	−0.9616^*^	0.1167	0.0755
WPV → SS → JB	− 0.1918^*^	−0.0949^**^	0.2527^**^	0.0182^*^	(0.0041, 0.0362)	0.2709^**^	0.0304	0.1039
WPV → SS → TI	−0.2422^**^	−0.0960^**^	0.3092^**^	0.0233^*^	(0.0078, 0.0416)	0.3325^**^	0.0386	0.0811

*N* = 1257; First path was controlled for age, marital status, professional title, employment form, department, years of experience, and daily working hours; second path was controlled for age, level of education, marital status, department, and daily working hours; third path was controlled for age, level of education, professional title, department, years of experience, and daily working hours; the displayed effects are standardized; WPV, workplace violence; JS, job satisfaction; JB, job burnout; TI, turnover intention; SS, social support; ^*^statistical significance level of *p* < 0.05, ^**^statistical significance level of *p* < 0.01, the indirect effect is significant (^*^) when the 95% CI does not include 0; SE, bootstrap regression standard error; R^2^, variance accounted for; c’, direct effect; a*b, indirect effect; c, total effect

## Discussion

### Prevalence of WPV against Chinese physicians

A cross-sectional study based on hospital physicians found that the prevalence of WPV experienced by physicians in the past year was 66.19%, which was lower than the result of a large sample of physicians experiencing WPV in China [19]. The prevalence of violence in hospitals in different countries varies [7–10]. This may be due to cultural differences in the perception of WPV in different countries and the diversity of assessment scales used in different studies. The prevalence of physicians exposed to verbal violence was 65.31%, which is the most frequent type of WPV experienced by Chinese physicians suffering from WPV. This result was similar to the previous study [2, 17, 19]. In addition, the prevalence of physical violence and sexual harassment/violence was 12.57 and 0.88%, respectively. These results indicated that the risk of Chinese physicians being exposed to WPV is high, which may be due to the tension between physicians and patients in China. In 2009, the Chinese government issued opinions on deepening the reform of the medical and health system; this round of health system reform was called the new medical reform [49]. It is worth noting that the new medical reform devotes more attention to the development of primary health care services, with the intention of maintaining the health of all citizens at the lowest possible cost [49, 50]. Although the new medical reform has achieved significant success, there are still some problems, such as the unbalanced allocation of health resources [51]. In China, due to an imbalance in the distribution of medical resources, health professionals are concentrated in public tertiary hospitals [51]. In addition, residents have the right to choose their own hospital, and the majority of residents initially selected for a tertiary hospital, which may cause more patients to go to the tertiary hospitals, thus increasing the workload of doctors, increasing the demands on their time with the technical problems of medical services and prompting a situation in which the humanistic care entailed in the service process is not provided, which is also important [2]. For a long time, the continual accumulation of contradictions between physicians and patients may have increased distrust on both sides, thus aggravating the tension between physicians and patients [52]. Therefore, the occupation of medical physician has become one of the most dangerous professions in China. The study also found that the more frequently physicians experience different types of violence in hospital settings, the greater the damage they may suffer, which is consistent with the results of Sun et al. [19]. The results also showed that the main perpetrators were patients’ relatives, followed by patients, which was similar to previous studies [19, 24].

### The negative impact of WPV on doctors’ workplace behavior

Research showed that for physicians who have experienced WPV on a frequent basis, job satisfaction is lower, job burnout is higher, and some physicians even leave their job, which may cause a shortage in hospital physicians. These findings are consistent with previous research results [2, 23, 26, 28]. WPV causes doctors to be in an unhealthy environment, which may lead to a marked decline in their enthusiasm. Moreover, they feel their own work does not receive respect and recognition from their patients and the patients’ families, and they begin to doubt their own value and professional status in the process of providing a medical service. Physicians with a higher frequency of violence may also have less empathy, which may lead to a lack of trust between physicians and patients. Eventually, it may cause them to avoid actively responding to and dealing with the conflicts they encounter, resulting in a lack of initiative in their work. Therefore, building a harmonious medical environment and reducing the prevalence of WPV is one effective measure for increasing physicians’ job satisfaction, reducing job burnout and turnover intention, and stabilizing the physicians’ team.

### The mediating effect of social support on physicians’ job satisfaction, job burnout, and turnover intention

Our research also showed that social support plays a mediating role in the WPV impact on the physicians’ workplace behavior (job satisfaction, job burnout, turnover tendencies), which was consistent with the results of previous studies [32, 33]. Moreover, the results showed that a* b * c′ was positive and can be considered as a supplementary mediation, indicating that part of the mediating role of social support was established. But the difference is that the direct and indirect effects of Paths 2 and 3 are opposite to those of Path 1. Obviously, these findings correspond to the results of correlation analysis. Physicians have a high level of work pressure, high risks, and heavy workloads, leading to long-term mental and physical fatigue. Once a doctor is attacked or experiences WPV, physicians’ negative emotions increase and job satisfaction declines, which may even cause indifference, apathy, helplessness, disappointment, lack of motivation, or resignation.

It is noteworthy that WPV is negatively correlated with social support. A previous study showed that the Deterioration Model of Social Support has been useful in discriminating the potential of stressors to reduce support [53]. This phenomenon may be due to a WPV -induced erosion of perceived social support for the increased danger of workplace behaviors among both primary and secondary victims; the loss of perceived social support also mediated workplace behaviors consequences. However, the Deterioration Deterrence Model of Social Support, which is similar to support mobilization models, has been used to explain how the perceived deterioration of social support can be counteracted by higher levels of received social support [54, 55]. If post-WPV support mobilization is implemented, WPVshould be positively correlated with received support.

The social relationships established by physicians in their work are complicated, involving the physician–patient relationship, physician–nurse relationship, physician –physician relationship, and physician–leadership relationship. When physicians suffer violence in the workplace, the importance of their social network is highlighted, and colleagues and leaders take positive measures in a timely manner (such as giving a warning and preventing the perpetrators from continuing the attack) to reduce the harm damage to doctors caused by WPV. At the same time, physicians can also receive encouragement and support from their family members, friends, colleagues, and leaders, and they are able to experience significant emotional support. These effective social supports will help reduce the physicians’ sense of job burnout and work indifference, enhance their personal sense of accomplishment, increase job satisfaction, and reduce turnover intentions. Therefore, social support plays an important role in alleviating the physical and mental health of WPV and the impact of WPV on workplace behavior.

Hospitals can establish a “code green” response team to manage any potentially violent situation. Preventive measures for WPV among physicians include increasing the awareness of potentially violent patients; wearing suitable clothes; maintaining proper positioning when communicating with patients; keeping a safe distance; maintaining the correct posture; and listening actively. We further advise hospitals to strengthen the training and management of physicians to reduce the harm to them from WPV. Hospitals could provide post-WPV support, reducing the impact of physicians’ psychological and workplace behaviors. Therefore, it is necessary to establish a unified and appropriate reporting system and provide training programs for health professionals.

### Limitations

Although there were some significant findings in the current study, several limitations still remain. First, purposive sampling results are greatly influenced by the preconceptions of the researchers. To the extent that subjective judgement may be biased, this can readily lead to sampling bias and cannot provide complete confidence in the results of the overall investigation. Second, we collected data about whether physicians had experienced WPV over the previous 12 months, so there may have been recall bias in the results. Third, the cross-sectional study reveals the status of the research object at a certain time, or the relationship between different variables at one point in time, and does not explain the causal relationship between the variables. However, this study has important significance for hospital managers as a reference, to maintain the stability of human resources.

## Conclusions

The results of this study showed that there is a high prevalence of WPV against physicians in tertiary hospitals. It is becoming increasingly important to deal with WPV in tertiary hospitals. To prevent and manage WPV, it is necessary to establish a unified and appropriate reporting system and provide training programs for health professionals. The effects of social support on workplace behaviors suggest that it has practical implications for interventions to promote the stability of physicians’ team. Moreover, it provides a good reference for hospital management and policy-making.

## Additional file

Additional file 1: Final Version of Questionnaire. (DOCX 25 kb)

## Data Availability

The datasets used and/or analysed during the current study are available from the corresponding author on reasonable request.

## Abbreviations

JB: Job burnout
JS: Job satisfaction
MBI-GS: Maslach Burnout Inventory-General Survey
MSQ-SS: Minnesota Satisfaction Questionnaire Short Scale
SS: Social support
SSRS: Social Support Rating Scale
TI: Turnover intention
WHO: World Health Organization
WPV: Workplace Violence
