# Supplementary material for: The impact of workplace violence on job satisfaction, job burnout, and turnover intention: the mediating role of social support
Source: Health Qual Life Outcomes. 2019 May 30;17:93. doi: 10.1186/s12955-019-1164-3 (PMC6543560; doi:10.1186/s12955-019-1164-3)
Supplement: Supplementary file 1 — Final Version of Questionnaire. (DOCX 25 kb) [file 12955_2019_1164_MOESM1_ESM.docx]

**Final Version of Questionnaire**

Respected physicians:

Hello! In recent years, many physicians experienced workplace violence. The purpose of this study is to understand the impact of workplace violence on workplace behavior.

Fill in the Description: Please in the appropriate box or number of √, some questions can be selected, part of the problem you fill in, please fill in the " " with patience.

***The information is only used for the subject research, absolute confidentiality, please rest assured that fill in. ***

**Part1：Please indicate your information**

Q1.Gender: Male□ Female□

Q2.Age: ≤30years□ 31-40years□ 41-50years□ 51-60years□ ＞60years□

Q3.Level of education: ＜Bachelor□ Bachelor□ ≥Master□

Q4.Marital status: Married□ Single□ Other□

Q5.Professional title: Junior□ Intermediate□ Senior□

Q6.Employment form: formal employee□ temporary employee□

Q7. Department: Emergency□ Outpatient□ Inpatient Department□

Specific: Medicine□ Surgical□ Gynaecology □ Pediatric□ Stomatology□ Features□ Auxiliary Inspection Department□ Other□

Q8. Years of Working: ＜1years□ 1-4years□ 5-10years□ 11-20years□

＞20years□

Q9. Working hours in the hospital: every day□ Less than 8 hours□ 8-10 hours□ 10-12 hours□ More than 12 hours□

**Part2: In the past 12 months, did anyone in the hospital perform the following behavior to you?**

**Workplace Violence Scale**

| **Types of violence** | **No** | **1 times** | **2 -3times** | **>3 times** |
| --- | --- | --- | --- | --- |
| Verbal violence |  |  |  |  |
| 1.Scold, abuse, abusive, demeaning or other detrimental to the dignity of the individual words.(face to face, telephone, letter or leaflets, micro-network, etc). |  |  |  |  |
| 2. Threats: threats related to personal and property safety, threatening complaints, trailing tracking and so on. |  |  |  |  |
| Physical violence (Attack by physical contact or with something, including hitting, kicking, slapping, pricking, pushing, biting, throwing, twisting arms, pulling hair, etc.) |  |  |  |  |
| 3.Physical violence do not cause physical damage |  |  |  |  |
| 4.Causing mild injury, such as pain, abrasions,  scratches, or ecchymosis. |  |  |  |  |
| 5. Resulting in significant injury, such as wounds,  fractures, visceral or head injury |  |  |  |  |
| 6.Causing serious consequences, such as  dysfunction or permanent disability |  |  |  |  |
| Sexual harassment/violence |  |  |  |  |
| 7. Sexual harassment or sexual provocation, including language, movement or exposure of sexual organs. |  |  |  |  |
| 8.Sexual assault, such as by the opposite sex to pull,  hold, kiss or touch the sensitive parts. |  |  |  |  |
| 9.Rape or attempted rape |  |  |  |  |

**Part3：Minnesota Job Satisfaction Questionnaire Revised Short Version**

| **Each item below is a comment on one aspect of your work. Please make a choice based on your satisfaction with all aspects of the work described below.** | **Very**  **Satisfaction** | **Satisfaction** | **general** | **Dissatisfied** | **Very**  **Dissatisfied** |
| --- | --- | --- | --- | --- | --- |
| 1.Always maintain a busy state |  |  |  |  |  |
| 2.Opportunities for independent work |  |  |  |  |  |
| 3.Now and then, there is a chance to do something different |  |  |  |  |  |
| 4.Opportunity to become an important player in the team |  |  |  |  |  |
| 5.The way my leaders treat their subordinates |  |  |  |  |  |
| 6.My superiors’ ability to make decisions |  |  |  |  |  |
| 7.Be able to do things that do not violate your conscience |  |  |  |  |  |
| 8.Stability of work |  |  |  |  |  |
| 9.The chance to do something for other people |  |  |  |  |  |
| 10.Tell people what to do |  |  |  |  |  |
| 11.The opportunity to give full play to one's abilities |  |  |  |  |  |
| 12.How to implement the Organization policy |  |  |  |  |  |
| 13.My pay and my workload |  |  |  |  |  |
| 14.Opportunities for promotion |  |  |  |  |  |
| 15.I'm free to make my own decisions. |  |  |  |  |  |
| 16.A chance to try my own way at work |  |  |  |  |  |
| 17.Working conditions |  |  |  |  |  |
| 18.Ways to get along with co-workers |  |  |  |  |  |
| 19.The rewards of my work done well |  |  |  |  |  |
| 20.The sense of accomplishment I get from my job |  |  |  |  |  |

**Part4：Social Support Scale**

Please read each topic carefully, in the most can express your true feelings that number above "√"

1. How many close friends do you have that can be supported and helped? (Choose only one)

A None of them B 1 to 2 C 3 to 5 D 6 or more

1. In the past year, you: (choose only one)

A Keep away from your family and live in a single room.

B Residences are constantly changing and spend most of the time with strangers.

C Living with classmates, colleagues or friends

D Live with your family

3.You and Neighbors: (Choose only one)

A Never care about each other, just nodding acquaintances

B You may be a little concerned when you encounter difficulties.

C Some neighbors are very concerned about you.

D Most neighbors care about you.

4. You and your colleagues: (choose only one)

A Never care about each other, just nodding acquaintances

B You may be a little concerned when you encounter difficulties.

C Some colleagues are very concerned about you.

D Most colleagues care about you.

5. Support and care from family members (in the appropriate box "**√**")

| **Project** | **No** | **very little** | **general** | **full support** |
| --- | --- | --- | --- | --- |
| Husband or wife (lover) |  |  |  |  |
| Parents |  |  |  |  |
| Children |  |  |  |  |
| Brothers and sisters |  |  |  |  |
| others |  |  |  |  |

6.In the past, when you were in a difficult situation, the sources of financial support and help in solving practical problems were as follows:

1) No source

2) The following sources: (multiple options):

A Spouse B Other family members C Friend D Relative E Colleague F Work unit

G Official or semi-official organizations such as party, league, trade union, etc.

H Non-official organizations such as religious and social groups

I Other (please list)

7. In the past, when you were in an emergency situation, the sources of comfort and concern you received were:

1) No source

2) The following sources: (multiple options):

A Spouse B Other family members C Friend D Relative E Colleague F Work unit

G Official or semi-official organizations such as party, league, trade union, etc.

H Non-official organizations such as religious and social groups

I Other (please list)

8. The way you talk when you are in trouble: (Choose only one)

A Never tell anyone B Reporting only to one or two people who are very close to each other

C If a friend asks you, you will say it.

D Actively complain about your troubles in order to gain support and understanding

9. How do you ask for help when you are in trouble: (Choose only one)

A Depend on yourself and don't accept help from others.

B Seldom seek help from others

C Sometimes I ask for help from others.

D When I am in trouble, I often turn to my family, relatives and organizations for help.

10. For organizations (such as Party and League organizations, religious organizations, trade unions, student unions, etc.) to organize activities, you: (only choose one)

A never participate in B occasionally participate C often participate D actively participate and activities

**Part5：Burnout Scale**

According to your own feelings and experience, judge them in your unit or the frequency of your body, and in the appropriate number to delimit √.

| **Project** | **Never** | **Very**  **Few times a year or less** | **Occasionally one months or less** | **Often one months a few times** | **Frequent every week** | **Very frequent**  **A few times a week** | **Every day** |
| --- | --- | --- | --- | --- | --- | --- | --- |
| 1.Work makes me feel exhausted |  |  |  |  |  |  |  |
| 2.I feel exhausted from work. |  |  |  |  |  |  |  |
| 3.I feel very tired when I get up in the morning and have to face the day's work. |  |  |  |  |  |  |  |
| 4.It's really stressful for me to work all day. |  |  |  |  |  |  |  |
| 5.Work makes me feel like I'm going to crash |  |  |  |  |  |  |  |
| 6.Since I started this job, I'm getting less interested in my job. |  |  |  |  |  |  |  |
| 7.I'm not as enthusiastic about work as I used to be. |  |  |  |  |  |  |  |
| 8.I doubt the meaning of my work. |  |  |  |  |  |  |  |
| 9.I'm more and more indifferent to my work. |  |  |  |  |  |  |  |
| 10.I can effectively solve the problems in my work. |  |  |  |  |  |  |  |
| 11.I think I'm making a useful contribution to the hospital. |  |  |  |  |  |  |  |
| 12In my opinion, I'm good at my job. |  |  |  |  |  |  |  |
| 13.I feel very happy when I finish some things at work |  |  |  |  |  |  |  |
| 14.I've done a lot of worthwhile work. |  |  |  |  |  |  |  |
| 15.I am confident that I can do all the work effectively. |  |  |  |  |  |  |  |

**Part6：turnover intention Scale**

1.Are you considering quitting your current job?

A :Often B:Occasionally C:Very little D:Never

2. Do you want to look for other jobs of the same nature?

A:Often B:Occasionally C:Very little D:Never

3. Do you want to look for other jobs of different nature?

A:Often B:Occasionally C:Very little D:Never

4. In your current situation and conditions, what do you think is the possibility of finding a suitable position in another organization?

A:Most possible BIt’s possible C:impossible D:Very impossible

5. If you know of another organization that has a job vacancy for you, what is the likelihood that you will get the job?

A:Most possible B:It’s possible C:impossible D:Very impossible

6. Will you quit your present job?

A:Will definitely B:Might be C:May not D:Definitely not.

**Thank you very much for your assistance, please check the above items are not**

**missing fill !**
